# The epidemiology of adverse drug events and medication errors among psychiatric inpatients in Japan: the JADE study

**DOI:** 10.1186/s12888-016-1009-0

**Published:** 2016-08-30

**Authors:** Nobutaka Ayani, Mio Sakuma, Takeshi Morimoto, Toshiaki Kikuchi, Koichiro Watanabe, Jin Narumoto, Kenji Fukui

**Affiliations:** 1 Department of Psychiatry, Graduate School of Medical Science, Kyoto Prefectural University of Medicine, 465 Kajii-cho, Kawaramachi-Hirokoji, Kamigyo-ku, Kyoto, 602-8566 Japan; 2 Department of Clinical Epidemiology, Hyogo College of Medicine, 1-1 Mukogawa, Nishinomiya, Hyogo 663-8501 Japan; 3 Department of Neuropsychiatry, Kyorin University School of Medicine, 6-20-2 Shinkawa, Mitaka, Tokyo, 181-8611 Japan

**Keywords:** Adverse drug event, Medication error, Epidemiology, Psychiatry, Patient safety

## Abstract

**Background:**

Knowledge of the epidemiology of adverse drug events (ADEs) and medication errors in psychiatric inpatients is limited outside Western countries. The nature of ADEs and medication errors are important for improving the quality of care worldwide; therefore, we conducted the Japan Adverse Drug Events Study, a series of cohort studies at several settings in Japan.

**Methods:**

This report included 448 inpatients with 22,733 patient-days in a psychiatric hospital and psychiatric units at a tertiary care teaching hospital over 1 year. Four psychiatrists and two other physicians reviewed all medical charts and related documents to identify suspected incidents. The physicians later classified those incidents into ADEs, potential ADEs, medication errors, or exclusions and evaluated the severity and preventability if the incidents were events.

**Results:**

During the study period, we identified 955 ADEs and 398 medication errors (incidence: 42.0 and 17.5 per 1000 patient-days, respectively). Among ADEs, 1.4 %, 28 %, and 71 % were life-threatening, serious, and significant, respectively. Antipsychotics were associated with half of all ADEs. The incidence of medication errors was higher in medical care units than in acute and nursing care units (40.9, 15.6, and 17.4 per 1000 patient-days, respectively). The monitoring and ordering stages were the most common error stages (39 % and 34 % of all medication errors, respectively), and 76 % of medication errors with ADEs were found at the monitoring stage. Non-psychiatric drugs were three times as likely to cause ADEs with errors compared to psychiatric drugs.

**Conclusions:**

Antipsychotic use, inadequate monitoring, and treatment of physical ailments by psychiatrists may contribute to the high incidence of medication errors and ADEs among psychiatric inpatients in Japan. Psychiatrists should be cautious in prescribing antipsychotics or unfamiliar medications for physical problems in their psychiatric patients, and should monitor patients after medication administration.

## Background

Adverse drug events (ADEs) are drug-related injuries resulting from medical intervention [1–3]. ADEs are generally the most frequent cause of injuries due to medical care in hospitals [4, 5]. Psychiatric inpatients are at high-risk for these injuries because pharmacotherapy plays a central role in psychiatric treatment [6, 7]. In addition, many psychiatric patients present with comorbid medical disorders that require treatment with non-psychiatric drugs, and when these conditions are treated in psychiatric hospitals, this puts patients at further risk for ADEs and medication errors [7, 8].

There is a need for more epidemiological data concerning appropriate medication use in order to provide safer and more effective pharmacological treatment for psychiatric inpatients. Previous studies, however, have noted the complexities of identifying ADEs and medication errors in psychiatric settings because it is difficult to distinguish ADEs caused by drugs from symptoms related to mental disorders; in addition, it can be difficult to define medication errors in these settings, as psychiatric pharmacotherapy often deviates from standard treatment [9, 10]. In fact, there have been notably few comprehensive studies on this topic, especially regarding ADEs [7, 11–13]. Furthermore, the studies that have been conducted all took place in Western countries, meaning that their results cannot be generalized to clinical settings in other countries without first assessing local data [14], because mental health services differ between countries. For example, longer hospital stays and lower staff ratios are two characteristics of Japanese psychiatric care [15], while many African countries suffer from a critical lack of psychiatrists and pharmacists [16]. To this end, we conducted a historical cohort study in psychiatric settings to estimate the incidence and nature of ADEs and medication errors among psychiatric inpatients in Japan.

## Methods

### Study design and patient population

This historical cohort study was conducted as part of a multicenter cohort study known as the Japan Adverse Drug Events (JADE) Study [17, 18]. As part of the JADE study series, we collected information using the standard JADE protocol. [3, 17, 18] Data were collected from the psychiatric inpatient units at one psychiatric hospital and one tertiary care teaching hospital. There were a total of 438 psychiatric inpatient beds between these two hospitals, including beds in acute care units, nursing care units, and medical care units. The acute care unit comprises the main section of a psychiatric department in which patients with an acute mental disorder receive targeted mental care. Psychiatric patients who have recovered from the acute stage of their condition but who still require nursing care are admitted to nursing care units. Medical care units are specialized sections within a psychiatric department that provide treatment to psychiatric patients with physical medical conditions. Both hospitals included in this study used electronic medical records.

At the tertiary care teaching hospital, patients were treated both by attending psychiatrists and by resident psychiatrists, who have <3 years of training after obtaining their medical license. Resident psychiatrists practiced under the supervision of attending psychiatrists and primarily ordered medications. In contrast, most of the psychiatrists at the psychiatric hospital were attending psychiatrists. Both hospitals admitted patients to the acute care or medical care units within the psychiatry department if psychiatric disorders were the main presenting problem and the patients’ physical problems were considered to be mild; internists provided medical consultations as needed. Conversely, if patients’ physical complications were considered to be more severe than their psychiatric problems, or if patients required intensive care (for example, as a result of myocardial infarction or femoral fracture, or if they required intubation), they were discharged from the psychiatric department and transferred to non-psychiatric wards for subsequent care.

Data were collected from all psychiatric inpatients who were admitted to and discharged from the acute, nursing and medical care units from April 1, 2010 through March 31, 2011. The main measures that were evaluated were patient-days and the number of admissions. The study was approved by the institutional review boards of the Kyoto Prefectural University of Medicine and by the institutional review boards of the two participating hospitals. The need for informed consent was waived because all data were collected as part of the hospitals’ daily practices.

### Definitions

The primary outcome measured in this study was the number of ADEs, defined as drug-related injuries resulting from medical intervention [1, 2]. The term ADE has a wide spectrum of definitions, including harm caused by drugs at a usual dosage (adverse drug reactions: ADRs) or at an unusual dosage, and also including harm from dose reduction and discontinuation of drug therapy [19]. For example, an extrapyramidal symptom, such as akathisia, occurring after a patient receives antipsychotics, and with no other apparent cause, is considered to be an ADE. Rebound insomnia that occurs following discontinuation of sedatives is another example of an ADE. An ADE was then categorized by severity as fatal, life-threatening, serious or significant. Fatal ADEs were those that resulted in death. Life-threatening ADEs were those that caused such issues as respiratory depression or suicidal behavior. Serious ADEs included gastrointestinal bleeding, falls, or a decrease in blood pressure. Significant ADEs included cases with milder symptoms, such as diarrhea, constipation, extrapyramidal symptoms or drowsiness.

A secondary outcome that was measured in this study was medication errors. Medication errors could occur at any step of the medication use process (ordering, transcribing, dispensing, administering or monitoring), and medication errors may or may not cause ADEs. If a medication error was found, the type of error and the stage in the process where it occurred were classified. The medication use process included the following stages: ordering by psychiatrists or other physicians; transcription by nurses; dispensing by pharmacists (or by psychiatrists and nurses, as was the case during the night shift and on weekends in the psychiatric hospital); administration by nurses or by patients; and monitoring by psychiatrists, other health professionals or by patients themselves.

ADEs were categorized as either preventable or non-preventable. An ADE was considered to be preventable if it resulted from a medication error or was otherwise ameliorable by available means (e.g., switching to a different drug or cautious monitoring after administration). An ADE that occurred in the absence of a medication error was defined as a non-preventable ADE. For example, a rash that occurred due to lamotrigine use in a patient without a history of lamotrigine-induced rash would not be considered a preventable ADE, but it would be considered as a preventable ADE if the patient had a history of such a rash.

We also classified ADEs according to their potential for causing injury. A potential ADE was an error that had the potential for injury but did not actually result in injury, either because of specific circumstances, chance, or because the error was intercepted. For example, if hypnotics were administered several hours earlier than prescribed, this would constitute a medication error and potential ADE, even if no negative effects were observed because hypnotics may cause immediate somnolence. On the other hand, early administration of anti-dementia drugs would be classified as a medication error but not a potential ADE because the drug rarely causes acute side effects.

### Data collection and classification

The definitions and methods used in this study were consistent with those from prior studies on this topic [3, 17, 18]. In this study, four psychiatrists and two physicians, all with experience in the classification of ADEs as a result of previous research on this topic, reviewed all patient charts from each participating hospital, along with laboratory results, incident reports and prescription queries. Research assistants used patient charts to compile demographic characteristics and administrative data for all enrolled patients in the cohort.

Once all data were collected from participating hospitals, the reviewers independently classified relevant incidents as an ADE, potential ADE or medication error, while also recording the details of those incidents. This included information about the name, dose, route and class of the drugs, the details of symptoms resulting from ADEs, and the details related to medication errors such as type, stage and persons who were in charge at the time the error occurred. The reviewers also independently classified all incidents according to their severity and preventability. After all suspected incidents were collected, the reviewers met to confirm the final classification for each incident. When the reviewers disagreed on the classification of an incident, they reached a consensus through discussion.

### Statistical analyses

The incidences per 1000 patient-days, crude rates per 100 admissions, and 95 % confidence intervals (CIs) were calculated as a whole and by unit types (acute care unit, nursing care unit, and medical care unit). Continuous variables are presented as means with standard deviations (SDs) or medians with interquartile ranges (IQRs), and categorical variables are shown as numbers and percentages. We used the χ^2^ test to assess the relationship between drug classes and preventable ADEs. We calculated inter-rater reliabilities using k statistics. Kappa scores between reviewers regarding the presence of an ADE were 0.96 (ADE v. potential ADE or exclude). The kappa for preventability was 0.95 (preventable v. non-preventable), while the kappa for severity was 0.43 (significant v. serious or life-threatening). These values were similar to those published in previous reports by Rothschild et al. (2007) and Morimoto et al. (2011). We performed all analyses using JMP V.11.2 (SAS Institute, Cary, North Carolina, USA) software.

## Results

There were a total of 448 admissions with 22,733 patient-days during the study period. The ages of the included patients ranged from 13 to 97 years old, and the mean age was 56 (SD 22) years. Forty-one (185/448) percent of patients were aged ≥65 years, and 247 (55 %) were female. The median hospital stay was 32 (interquartile range 15–75) days. The acute care, nursing care and medical care units admitted 341 (76 %), 75 (17 %), and 32 (7 %) patients, respectively (Table 1). Of all admissions, approximately 42 % were involuntary admissions. The most common reasons for admission were schizophrenic disorders and dementia, and the median number of medications patients were taking on admission was 6 (range 4–8) (Table 1).

**Table 1:** Demographic data for the study population

Factors	No. of patients
	Total (*n = 448*)
Age ≥ 65 years, *n* (%)	185 (41)
Female, *n* (%)	247 (55)
Admitting unit, *n* (%)	
Acute	341 (76)
Nursing	75 (17)
Medical	32 (7)
Admission pathway, *n* (%)	
Scheduled admission	247 (55)
Emergency admission	201 (45)
Nonresident physician in charge, *n* (%)	379 (85)
Involuntary admission, *n* (%)	186 (41.5)
Number of prescribed medications on admission, median (quartile)	6 (4–8)
Primary diagnosis,^a^ *n* (%)	
Dementia	97 (21.7)
Other organic disorders	19 (4.2)
Mental or behavioral disorder due to substance use	48 (10.7)
Schizophrenia and other psychotic disorders	113 (25.2)
Mood disorders	84 (18.8)
Depression	38 (8.5)
Mania, Bipolar disorder	32 (7.1)
Other mood disorders	14 (3.1)
Neurotic, stress-related and somatoform disorders	40 (8.9)
Anorexia	17 (3.8)
Mental retardation	11 (2.5)
Development disorder	12 (2.7)
Other	7 (1.6)

^a^Diagnoses based on the International Classification of Diseases, Tenth Revision [24]

### Adverse drug events

**Fig. 1:**
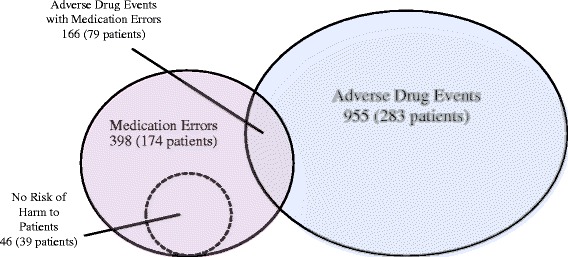
Relationship between adverse drug events and medication errors

We identified 1234 suspected incidents, and through reviews and discussions of these suspected incidents, we identified 955 ADEs among 283 patients (63 %) (Fig. 1). The incidence of ADEs was 42.0 [95 % CI 39.4–44.6] per 1000 patient-days, and the crude rate was 213 [95 % CI 184–243] per 100 admissions (Table 2). Significant ADEs accounted for 71 % (677 events in 263 patients) of all events, followed by serious ADEs (28 %, 265 in 124) and life-threatening ADEs (1.4 %, 13 in 12). There were no fatal ADEs that occurred during the study.

**Table 2:** Incidences of adverse drug events, medication errors and preventable adverse drug events

Unit	*n*	Patient-days	ADEs	Incidence^a^	95 % CI	Crude rate^b^	95 % CI
Acute	341	16834	725	43.1	40.0–46.1	213	179–246
Nursing	75	4480	157	35.0	29.7–40.4	209	144–275
Medical	32	1419	73	51.4	40.0–62.9	228	88.6–368
Total	448	22733	955	42.0	39.4–44.6	213	184–243
Unit	*n*	Patient-days	Medication Errors	Incidence^a^	95 % CI	Crude rate^b^	95 % CI
Acute	341	16834	262	15.6	13.7–17.4	76.8	62.0–91.7
Nursing	75	4480	78	17.4	13.6–21.2	104	56.3–152
Medical	32	1419	58	40.9	30.6–51.2	181	73.4–289
Total	448	22733	398	17.5	15.8–19.2	88.8	72.9–105
Unit	*n*	Patient-days	Preventable ADEs	Incidence^a^	95 % CI	Crude rate^b^	95 % CI
Acute	341	16834	86	5.1	4.0–6.2	25.2	16.4–34.1
Nursing	75	4480	38	8.5	5.8–11.2	50.7	17.5–83.8
Medical	32	1419	42	29.6	20.8–38.4	131	35.9–227
Total	448	22733	166	7.3	6.2–8.4	37.1	25.8–48.3

*ADEs* adverse drug events, *CI* confidence interval

^a^Per 1000 patient-days

^b^Per 100 admissions

The most common class of drugs associated with ADEs was atypical antipsychotics (34 %, 323/955), and almost half of ADEs (46.9 %, 448/955) were associated with typical and atypical antipsychotics. Non-psychiatric drugs accounted for 16 % (124/789) of non-preventable ADEs, but were associated with 42 % (69/166) of all preventable ADEs. In other words, the proportion of preventable ADEs to all ADEs associated with non-psychiatric drugs (69 per 193 ADEs; 36 %) was higher compared to psychiatric drugs (97 per 762 ADEs; 13 %) (*P* < 0.001) (Table 3).

**Table 3:** Frequency of adverse drug events according to drug class

Drug Class	ADEs, *n* (%) (*n = 955*)	Preventable ADEs, *n* (%) (*n = 166*)	Non-preventable ADEs, *n* (%) (*n = 789*)	Potential ADEs, *n* (%) (*n = 186*)	Intercepted potential ADEs, *n* (%) (*n = 23*)	Non-intercepted potential ADEs, *n* (%) (*n = 163*)
Antibiotics	10 (1.0)	0 (0)	10 (1.3)	2 (1.1)	0 (0)	2 (1.2)
Antihypertensives	14 (1.5)	3 (1.8)	11 (1.4)	7 (3.8)	1 (4.3)	6 (3.7)
Cardiovascular drugs	8 (0.8)	1 (0.6)	7 (0.9)	12 (6.5)	2 (8.7)	10 (6.1)
Anticoagulants	9 (0.9)	2 (1.2)	7 (0.9)	1 (0.5)	0 (0)	1 (0.6)
Antihyperlipidemics	1 (0.1)	0 (0)	1 (0.1)	0 (0)	0 (0)	0 (0)
Antidiabetics	10 (1.0)	2 (1.2)	8 (1.0)	9 (4.8)	1 (4.3)	8 (4.9)
Peptic ulcer drugs	1 (0.1)	0 (0)	1 (0.1)	0 (0)	0 (0)	0 (0)
Laxatives	40 (4.2)	10 (6.0)	30 (3.8)	7 (3.8)	0 (0)	7 (4.3)
NSAIDs	6 (0.6)	0 (0)	6 (0.8)	7 (3.8)	0 (0)	7 (4.3)
Antiallergic agents	2 (0.2)	0 (0)	2 (0.3)	0 (0)	0 (0)	0 (0)
Electrolytes or fluids	58 (6.1)	50 (30.1)	8 (1.0)	21 (11.3)	1 (4.3)	20 (12.3)
Chinese herbal medicines	2 (0.2)	0 (0)	2 (0.3)	0 (0)	0 (0)	0 (0)
Sedatives (benzodiazepine)	66 (6.9)	28 (16.9)	38 (4.8)	53 (28.5)	0 (0)	53 (32.5)
Sedatives (other)	15 (1.6)	4 (2.4)	11 (1.4)	5 (2.7)	1 (4.3)	4 (2.5)
Anxiolytics	31 (3.2)	6 (3.6)	25 (3.2)	5 (2.7)	2 (8.7)	3 (1.8)
Antidepressants (SSRI, SNRI, NaSSA)	58 (6.1)	2 (1.2)	56 (7.1)	4 (2.2)	3 (13.0)	1 (0.6)
Antidepressants (other)	62 (6.5)	6 (3.6)	56 (7.1)	1 (0.5)	1 (4.7)	0 (0)
Mood stabilizers	45 (4.7)	14 (8.4)	31 (3.9)	4 (2.2)	2 (8.7)	2 (1.2)
Antipsychotics (atypical)	323 (33.8)	32 (19.3)	291 (36.9)	34 (18.3)	6 (26.1)	28 (17.2)
Antipsychotics (typical)	125 (13.1)	4 (2.6)	121 (15.3)	1 (0.5)	0 (0)	1 (0.6)
Anticonvulsants	8 (0.8)	1 (0.6)	7 (0.9)	3 (1.6)	0 (0)	3 (1.8)
Anti-parkinsonian drugs	24 (2.5)	0 (0)	24 (3.0)	1 (0.5)	0 (0)	1 (0.6)
Anti-dementia medicines	5 (0.5)	0 (0)	5 (0.6)	1 (0.5)	1 (4.3)	0 (0)
Other drugs	32 (3.4)	1 (0.6)	31 (3.9)	8 (4.3)	2 (8.7)	6 (3.7)
Psychiatric drugs^a^	762 (79.8)	97 (58.4)	665 (84.3)	112 (60.2)	16 (69.6)	96 (58.9)
Non-psychiatric drugs^b^	193 (20.2)	69 (41.6)	124 (15.7)	74 (39.8)	7 (30.4)	67 (41.1)
All drugs	955 (100)	166 (100)	789 (100)	186 (100)	23 (100)	163 (100)

*ADEs* adverse drug events, *NSAIDs* nonsteroidal anti-inflammatory drugs, *SSRI* selective serotonin reuptake inhibitor; *SNRI* serotonin-noradrenaline reuptake inhibitor, *NaSSA* noradrenergic and specific serotonin antidepressants

^a^Psychiatric drugs include: sedatives (benzodiazepine), sedatives (other), anxiolytics, antidepressants (SSRI, SNRI, NaSSA), antidepressants (other), mood stabilizers, antipsychotics (atypical), antipsychotics (typical), anticonvulsants, anti-parkinsonian drugs and anti-dementia medicines

^b^Non-psychiatric drugs include: antibiotics, antihypertensives, cardiovascular drugs, anticoagulants, antihyperlipidemics, antidiabetics, peptic ulcer drugs, laxatives, NSAIDs, antiallergic agents, electrolytes or fluids, Chinese herbal medicines and other drugs

When ADEs were assessed by organ system, central nervous system symptoms (including falls, over-sedation and extrapyramidal symptoms) were the most frequent symptoms, accounting for 44 % (415/995) of all ADEs, followed by gastrointestinal symptoms (including diarrhea and constipation) (34 %, 326/955), allergic or skin symptoms (including drip leakage) (6 %, 58/955) and metabolic or liver dysfunction (5 %, 49/955).

### Medication errors and potential adverse drug events

We identified 398 medication errors among 174 patients (39 %). The incidence was 17.5 [95 % CI 15.8–19.2] per 1000 patient-days, and the crude rate was 88.8 [95 % CI 72.9–105] per 100 admissions. Among the 398 medication errors, 166 actually resulted in ADEs and were therefore classified as preventable ADEs, whereas 186 had the potential to cause injury but did not result in observed harm (Fig. 1). The incidence and crude rates were approximately two times higher in the medical care units compared to the other units. Furthermore, the incidence of preventable ADEs in the medical care units (29.6) was much higher compared to the acute care units (5.1) and nursing care units (8.5) (Table 2).

The incidence of preventable ADEs and non-preventable ADEs was 7.3 [95 % CI 6.2–8.4] and 34.7 [95 % CI 32.3–37.1] per 1000 patient-days, respectively. Thus, 17.4 % (166/955) of ADEs were considered preventable. The incidence of potential ADEs was 8.2 [95 % CI 7.0–9.4] per 1000 patient-days. Forty-six medication errors were determined to carry no risk of injury to patients, so these errors were not considered to be potential ADEs. Twelve percent of potential ADEs (23 cases) were intercepted before a drug was administered and were thus classified as intercepted potential ADEs. Medication errors were most frequently associated with the monitoring stage (39 %, 155/398) and ordering stage (34 %, 134/398) of treatment. In addition, 76 % (126/166) of preventable ADEs occurred during the monitoring stage. Potential ADEs occurred most frequently during the ordering stage, accounting for 46 % (86/186) of all potential ADEs, followed by the administering stage (36 %, 67/186).

## Discussion

We determined that ADEs and medication errors were common in Japanese psychiatric inpatient settings. ADEs were observed in 63 % of psychiatric inpatients with an incidence of 42 per 1000 patient-days, and medication errors were observed in 39 % of inpatients with an incidence of 17.5 per 1000 patient-days. Most of these ADEs were not preventable (83 % of ADEs), and 29 % of ADEs were classified as serious or life-threatening. In addition, we identified frequent medication errors at the monitoring stage (39 % of all medication errors), and this was more evident for preventable ADEs (76 % of all preventable ADEs occurred at this stage).

### Comparison with findings from previous studies in psychiatric settings

Although there have been several previous studies on ADEs (or ADRs) and medication errors in psychiatric settings, comparisons between the previous studies were difficult because they used different designs and denominators [20]. In addition, among studies utilizing the same denominator but with different study designs, there were significant differences in the reported rates of medication errors (e.g., 0.79 potential ADEs per 1000 patient-days based on a reporting system [21] vs. 1516 medication errors per 1000 patient-days on a retrospective chart review [8]). Therefore, in order to compare our findings with those of previous studies in different settings, we adopted the same definition and methodology used in the study performed by Rothschild et al., which took place in psychiatric settings in the USA [7], as well as those of other studies in general settings in the USA [2] and Japan [17]. In comparison with the present study, Rothschild et al. reported one-quarter incidence of ADEs (10 per 1000 patient-days) and one-third medication errors (6.3 per 1000 patient-days). The difference become even more evident regarding the crude rate of ADEs per 100 admissions (213 v. 10.2) and medication errors (88.8 v. 6.4); this is likely a result of the fact that the mean length of stay is much longer in Japan compared to the USA (50.7 v. 10.3 days).

The reasons for the higher incidence of ADEs in the present study may result from differences inpatient characteristics between this study and the USA study. The most common diagnosis in the USA study was mood disorders (66.4 %), while schizophrenic disorder (25 %) followed by dementia (22 %) were the most common disorders in the present study. In accordance with this finding, Schmidt et al. (1984) reported a similar rate of ADRs (346 per 100 admissions) in a previous study performed in Germany in which schizophrenic disorder was the most common diagnosis (37 %) [11], and Hermesh et al. (1985) reported that elderly patients with organic brain disorders were at high risk of ADRs [12].

Differences of the medical system in the treatment of physical complications in psychiatric inpatients may be another possible reason for the discrepancy between our findings and prior reports on this topic. Patients in psychiatric settings in Japan tend to receive more extensive treatments for physical complications compared to patients in the USA, where patients with severe physical complications are commonly transferred to a general-care setting, especially in cases that require electrocardiographic monitoring or a continuous intravenous drip [7]. As a result, patients in Japanese inpatient psychiatric units may be at higher risk of ADEs and medication errors, as prescribing unfamiliar drugs is associated with medication errors due to lack of experience and knowledge for practitioners in both psychiatric and general settings [7, 22]. In the present study, the proportion of preventable ADEs associated with non-psychiatric drugs was three times higher compared to psychiatric drugs (36 % v. 13 %, respectively), and the incidence of preventable ADEs was higher in the medical care units compared to acute and nursing care units (27.5 v. 5.1 v. 9.2 per 1000 patient-days, respectively).

### Comparison to general-care settings in Japan

Compared with a previous study on ADEs in general-care settings in Japan [17], we also found a higher incidence of ADEs (42.0 v. 17.0 per 1000 patient-days) and medication errors (17.5 v. 8.7 per 1000 patient-days). The higher incidence of ADEs and medication errors in psychiatry units may result from the specific complexities of the medications used to treat psychiatric patients. Our results demonstrated that almost half of ADEs were associated with antipsychotics, which is in accordance with previous studies that also found that antipsychotics were the drug class most frequently associated with ADEs [7, 11, 13]. Antipsychotics are prescribed for many patients—not only for the treatment of schizophrenia but also for sedation in agitated patients—and they may cause a wide range of ADEs, including neurological, gastrointestinal, cardiovascular, metabolic and endocrine symptoms. The frequency and intensity of ADEs resulting from the use of antipsychotics (especially when used at high dosages for patients with severe mental disorders) may contribute to the high incidence of ADEs in psychiatric units. In addition, psychiatric patients with severe mental disorders may lack self-awareness, and as a result, they may not be able to fully report their symptoms due to ADEs to medical staff. Furthermore, if they unexpectedly refuse to take their medications, this may cause more frequent medication errors. Finally, monitoring errors may occur due to a combination of lack of experience and knowledge regarding the management of physical complications on the part of psychiatrists as well as inadequate staffing in psychiatric units [15].

### Clinical implications

Psychiatrists usually regard ADEs like constipation from antipsychotics and drowsiness from sedatives as common and unavoidable consequences of medication, and believe that such ADEs seldom cause serious outcomes. However, serious ADEs are not rare, even though only a small percentage of ADEs are serious because ADEs occur frequently in medical care. According to the results of this research, life-threatening and serious ADEs accounted for 1.4 % (13 events in 12 patients) and 28 % (265 events in 124 patients) of events, respectively. Psychiatrists sometimes have to decide whether or not to continue administering medications associated with ADEs to treat patients with serious mental conditions; therefore, it is important to identify ADEs at an earlier stage to prevent serious events or to ameliorate their severity.

Moreover, as demonstrated by the results of the present study, psychiatrists were likely to make medication errors with ADEs during physical treatments, especially during the monitoring stage. This may be because psychiatrists focus on psychiatric problems and are less likely to treat physical problems, especially in psychiatric settings. Physicians usually tend to keep psychiatric inpatients at a distance, and psychiatrists in Japan may thus have to treat physical complications, with the exception of very severe physical conditions. Fragmentation of the physical and mental health systems is one of the barriers that hinders patients from receiving adequate care; [23] therefore, fixing the fragmented systems and increasing communication between physicians and psychiatrists could improve patients’ physical health and minimize injury from medications among psychiatric inpatients in Japan and other countries.

### Study limitation and strengths

Our study had several limitations. First, we conducted this study at one psychiatric hospital and one tertiary care teaching hospital. Therefore, our results may not represent other hospitals, although we attempted to mitigate this limitation by including both a psychiatric hospital and a tertiary care teaching hospital to represent a wide range of psychiatric settings. Second, we could not estimate the incidence and nature of ADEs and medication errors caused by doctors with other specialties in psychiatric settings because almost all medications were prescribed by psychiatrists in this study. Third, some ADEs and medication errors may have been missed, which would mean that our results underestimate the true incidence. However, we were able to precisely evaluate and collect data on confirmed incidents, especially physical symptoms due to ADEs; this was because internists with experience in the classification of ADEs as a result of previous research on this topic [17, 18] played a leading role in this study. In addition, more robust alternatives for measuring ADEs and medication errors have not yet been developed, and the approach we used is the approach that is currently used most widely, suggesting that the figures obtained in this study are the best that are currently available.

## Conclusions

We found high incidences of ADEs and medication errors in general psychiatric settings and identified some risk factors for ADEs, including prescription of antipsychotics and treatment during the monitoring stage after drugs are administered. Therefore, clinicians should be cautious in prescribing antipsychotics and while monitoring patients after administration, especially when patients are unable to report their symptoms due to a severe mental condition. Furthermore, because of the higher risk of ADEs and medication errors during the treatment of physical complications, consultation with physicians in other departments is essential when psychiatrists are considering prescribing unfamiliar medications for physical problems in their psychiatric patients.

## Abbreviations

ADE: Adverse drug event
CI: Confidence interval
IQR: Interquartile range
SD: Standard deviation
